# Post-partum depression, anxiety and marital satisfaction: A perspective from Southeastern Nigeria

**DOI:** 10.4102/sajpsychiatry.v24i0.1109

**Published:** 2018-03-22

**Authors:** Jaclyn I. Odinka, Marybasil Nwoke, JohnBosco C. Chukwuorji, Kenneth Egbuagu, Philip Mefoh, Paul C. Odinka, Kennedy U. Amadi, Rosemary C. Muomah

**Affiliations:** 1 Department of Psychology, Faculty of Social Sciences, University of Nigeria, Nigeria; 2 Department of Psychological Medicine, College of Medicine, University of Nigeria, Nigeria

## Abstract

**Background:**

Many studies have noted the high prevalence of post-partum depression (PPD) and anxiety associated with poor marital satisfaction, albeit amidst a dearth of literature on comorbid PPD and anxiety among women in Nigeria.

**Objective:**

The study was aimed to assess the prevalence of PPD and anxiety, and to investigate their relationship with marital satisfaction in low-risk women in Enugu, Southeastern Nigeria.

**Method:**

A cross-sectional study of 309 randomly selected nursing mothers at two tertiary health institutions. Socio-demographic Questionnaire, Hospital Anxiety and Depression Scale, and Index of Marital Satisfaction (IMS) were used to collect data on demography, anxiety and depression and marital relationship respectively. All statistical tests were performed at a significant level of 0.05.

**Results:**

The age range of the respondents was 20–46 years; mean and s.d. was 29.65 and ± 4.87, respectively, and most of them were graduates of tertiary educational institutions (74.1%). The prevalence of post-partum anxiety was 31.1% and of PPD was 33.3%. Marital dissatisfaction was observed in 39.5% (122) of the respondents who were mothers. Those with co-morbid depression and anxiety (22.0%) had worse marital dissatisfaction. The strongest correlation with depression and anxiety was item 12 of IMS (‘feel that my partner doesn’t confide in me’).

**Conclusion:**

There was a high prevalence of marital dissatisfaction, PPD and anxiety among nursing mothers in Enugu, but with low detection rate. The effects of PPD and anxiety on the mother, her marital relationship and her infant make them essential conditions for early diagnosis, prevention and treatments.

## Introduction

For most women having a baby, especially their first child, is a significant transitional life event.^1^ The first weeks and months postpartum may be associated with emotional upheaval.^2^ The nursing mother tries to meet the needs of her baby, to adapt to the changes in her sleep pattern and to recover from the process of delivery physically.^3^ Also, the depth of their feelings may be related to hormonal changes, fatigue and the pain of incisions, swollen breasts or sore nipples.^4^ Post-partum period involves changes in marital and family relationships and is commonly a cause of additional financial burden, even among households with relatively high income^5^ but worse among the low socioeconomic class.^6,7^

Post-partum depression (PPD) and anxiety affect approximately 10% – 20% of all mothers in Western societies.^8^ A prevalence of 23% – 31% was reported for PPD in Southeastern Nigeria,^9,10^ while Fatoye et al.^11^ reported that between 5% and 38% of women suffer from PPD in Southwestern Nigeria. The differences in the prevalence might be because of the effect of social, cultural, lifestyle and racial factors on depression.^12^ These statistics indicate that PPD might be a very common experience in Nigeria. Post-partum depression is related to the level of social support,^2^ previous depressive illness, stress, psychological factors and obstetric factors.^6,7^

Some psychosocial stressors in pregnancy may be because of cultural influence issues relevant to Igbo culture where marriage is patrilineal, with emphasis on begetting a child, especially a male child, to sustain a family lineage and to guarantee the position of the woman in the family.^13^ In Igbo culture and practices, not begetting a child, especially a male child, is one of the most relevant factors that could influence marital discord, and this could lead to marital dissatisfactions. Also, in Igbo culture and tradition, it is believed that a woman is not just married to a man but to the extended family, and for this, to a great extent, the in-laws have an influence on the marriage. Pressures from the extended family sometimes compound psychosocial stress postpartum. Psychosocial stressors in pregnancy or early postpartum is a strong predictor of post-partum depressive symptoms,^14,15,16,17^ and marital problems during pregnancy increase the risk of PPD and anxiety.^18,19,20^ However, the causal pathways between psychosocial stressors and depression may go in both directions.^17^

Post-partum depression can develop at any point during the first year of postpartum, with a peak of incidence in the first 4 months of postpartum.^21,22^ The mother may suffer an inability to take care of her new baby either emotionally or physically, which can impair early bonding and attachment process between mother and baby and could also hinder effective infant care skills^1^ and, if untreated, can affect the quality of mother-child relationship with deleterious effects on the child’s cognitive, psychosocial and physical development.^1,23^ PPD may also adversely affect the marital relationship.^23^

Depression often co-occurs with other disorders, especially anxiety symptoms.^24,25^ Some authors^26,27^ have also found associations between experiencing stressful life events and maternal depression and anxiety. Previous studies have shown high correlations between measures of depressive and anxiety symptoms and relationship adjustment.^25,28^ Braverman and Britton^29^ reported a significant positive association between post-partum anxiety, as well as depression and infant mobility, temperament, adaptability and sensibility. Generally, PPD and anxiety may have implications for marital satisfaction.

Marital satisfaction can be defined as a subjective experience of one’s personal happiness and contentment in a marital relationship.^30^ Greeff^31^ describes marital satisfaction as marital relationship in which couples have many agreements with each other on many variables such as family happiness, spouse’s satisfaction with the sexual relationship, satisfaction with general quality of life, family strengths, flexibility in the way free time is spent in the marital relationship, sound relationships with family and friends, conflict management and resolution, and communication within the marriage. The couple is satisfied with the type and level of their relations and the type and quality of leisure times, and has proper management of their finances and time.^31^

There is a significant relationship among PPD, quality of marital relationship and mental health.^32,33^ Previous community surveys have shown that marital adjustment is concurrently,^34^ and prospectively,^35^ associated with anxiety disorders, and those anxiety symptoms could predict a decline in marital adjustment over time.^36^ Lower marital adjustment is prospectively associated with an onset of anxiety disorders in non-pregnant mothers.^35^

Given the high prevalence of PPD and anxiety, their multifactorial nature and their association with poor marital satisfaction, and the importance of mental health promotion in women, any attempt to reduce any risk factors affecting their incidence can lead to a decreased rate of depression and anxiety. The development of the relevant framework is important to develop the type of strategies that could be put in place for the prevention, early detection of women at risk, and for the appropriate intervention programme to be instituted for such mothers by addressing possible risk factors such as poor marital satisfaction through active case findings, counselling and social support during antenatal visits, as well as increasing the resources available for mental health services.

The study on PPD and anxiety becomes more imperative in this part of the world, and more so given the dearth of information on their correlates in the sub-Saharan African settings, when compared to the Western world, and the importance of mental health promotion in women. It is pertinent to conduct empirical investigations of factors associated with these disorders, as any reasonable attempt to reduce any of the confirmed risk factors affecting the incidence of PPD and anxiety can go a long way in reducing the high prevalence. This study was aimed to assess the prevalence of PPD and anxiety and to investigate their relationship with marital satisfaction in low-risk women in Enugu, Southeastern Nigeria. These should be an urgent challenge for Nigerian public health officials.

## Method

### Study area

This study was carried out at the University of Nigeria Teaching Hospital (UNTH), Ituku-Ozalla, and Enugu State University of Science and Technology (ESUT) Teaching Hospital, both in Enugu, Enugu State, Southeastern Nigeria. The two hospitals, which are among the seven university teaching hospitals in Southeast Nigeria, provide medical services to residents of Enugu state and also receive referrals from and beyond all five southeastern states of Nigeria. Enugu is one of the most cosmopolitan Igbo cities and has the highest concentration of tertiary educational institutions in Southeastern Nigeria. Enugu is seen as a regional capital, as well as the Enugu State capital. Enugu State has a population of 3.3 million people of whom 95% are Igbo, and about 59% of the population live in the rural areas.^37^

Southeastern Nigeria (one of the six geopolitical zones or divisions in which Nigeria’s 36 federating units or states are grouped) is primarily inhabited by the people of the Igbo ethnic group, who speak Igbo and one of the three largest and most influential ethnic groups in Nigeria.^38^ The Igbos constitute 18% of the total Nigerian population of 170 million, that is, approximately 30 million.^39^ Igbo people are predominantly Christians.^38^

### Sample identification and recruitment procedures

Recruitment of participants was done at the postnatal clinics and Children’s welfare clinics of the two hospitals. All the nursing mothers who came for follow-ups and immunisation for children, between November and December 2015, formed the sampling frame. With a prevalence of 23%^9^ and an absolute standard error of 0.05 and a standard normal variance of 1.96, data collection was performed until the sample size of 309, considered adequate for the study, using the appropriate Cochran’s sample size formula for proportions.^40^ With the help of the outpatients’ attendance list, the mothers were randomly selected, using a table of random numbers. A total of 334 women were chosen for preliminary interviews to confirm their personal history, medical history, past psychiatric history, obstetric history and life events, and 309 who met the inclusion criteria were recruited into the study. The assessment was done from 6th week to 14th week of postpartum (the period from 2nd to the 4th round of immunisation in Nigeria), as the highest prevalence has been observed within this time.^22,41^ Very minimal assistance was given to the mothers as they were literate enough to complete the questionnaires.

### Ethical issues

First, the mothers were given a complete description of the study by one of the researchers, before a written informed consent was sought and obtained from them. The mothers who gave their consent were later given the three self-administered questionnaires to complete.

They were told the average time it could take to complete the questionnaires and were given enough privacy. The questionnaires were anonymously completed, as they were also assured of confidentiality.

### Inclusion or exclusion criteria

All the women that delivered their babies from the gestational age of 36 weeks were included in the study. Also, all women with conditions that might decrease the reliability of the instrument for the assessment of PPA and PPD, such as multiple births, obstetric and pregnancy complications, major chronic disease, any severe or unstable medical illness, a current or previous history of psychiatric disorders, and recent traumatic and life events, were excluded from the study. This information was through self-report during preliminary interviews.

### Instruments

#### Socio-demographic questionnaire

Socio-demographic questionnaire was used to collect socio-demographic data such as age, the number of children, marital status, the area of residence (rural or urban), and educational attainment.

#### Hospital Anxiety and Depression Scale

Hospital Anxiety and Depression Scale (HADS) is a self-report questionnaire^42^ and was developed to detect adverse anxiety and depressive states among outpatients in non-psychiatric hospital clinics. Some of its positive qualities include brevity and easiness of use, excellent reliability and validity, as well as efficiency in screening and case-finding.^43^ It has also been found useful in the assessment of probable psychiatric morbidity in the community. Hospital Anxiety and Depression Scale consists of seven items each for depression and anxiety, and the scores are rated on a four-point scale ranging from 0 to 3, with 21 as the highest score. For the stringent and robustness of case-findings, a cut-off point of 11 and above in either or both of the anxiety and depressive subscales indicates depression and anxiety disorder based on the DSM-IV and ICD-10 diagnostic criteria for current depressive disorder or anxiety disorder, while between 8 and 10 on both subscales indicates borderline case. It has been validated in many countries, among which is Nigeria.^44^

Hospital Anxiety and Depression Scale was not designed to be a clinical diagnostic tool but screening tool,^45^ and its use in this study was as a screening tool to flag probable depressive and anxiety illness and was usually a follow-up with a psychiatric interview in the psychiatric clinic for definitive diagnosis and treatment for women that needed such intervention. Therefore, its use in this study was to flag up probable depressive and anxiety illness and did not necessarily confer a diagnosing of depression and anxiety. However, HADS has also been found to perform well in assessing the severity in already diagnosed people with depression or anxiety.^46^

#### Index of Marital Satisfaction

Index of Marital Satisfaction (IMS) is a 25-item inventory, designed by Hudson,^47^ that measures the degree and severity or magnitude of the problems one spouse or partner perceives to be having in the marital relationship with his or her partner. The focus is on current problems that have reduced marital satisfaction. There is a direct scoring (items 2, 4, 6, 7, 10, 12, 14, 15, 18, 22, 24, 25) and reverse scoring (1, 3, 5, 8, 9, 11, 13, 16, 17, 19, 20, 21, 23) of the items. The final score is obtained by adding together the result of the direct scores and the reverse scores to obtain the client’s raw score. The IMS was derived from subtracting 25 from the raw total score. A score below 30 was indicative of satisfaction with the relationship, while a score above 30 was indicative of dissatisfaction. Hudson^47^ reported a Cronbach’s alpha of 0.96 and two-week intervals of test-retest reliability of 0.96. It has been validated in many countries, and the internal consistency has been established in Nigeria.^48^

### Data analysis

The raw data were keyed into Epidata software version 3.1 (The EpiData Association, Odense, Denmark) for data entry, documentation and storage, and was later transported to Statistical Package for the Social Sciences (SPSS) version 20.0 (IBM, USA) for analysis. The descriptive frequency distribution of the socio-demographic characteristics of the nursing mothers, the distribution of the mothers’ anxiety and depression status and the distribution of the mothers’ co-morbidity for anxiety and depression were determined. Chi-square test was used to assess for an association between socio-demographic data and scores of IMS, and mothers with and without PPD and PPA, respectively. As scores on the IMS (continuous variables) did not have a Gaussian distribution, Spearman’s correlations were performed to test the relationships between the Marital Satisfaction (independent variables) and the post-partum anxiety and depression (dependent variables). All statistical tests were two-sided and were performed at a significant level of 0.05.

## Ethical consideration

The approval for the study was sought and obtained from the institutional review board (IRB) of the College of Medicine, University of Nigeria Enugu Campus, Enugu, Nigeria. The study has, therefore, been performed in accordance with the ethical standards laid down in the 1964 Declaration of Helsinki and its later amendments.

## Results

Table 1 shows the distribution of the mothers according to age range, marital status, educational status, the area of residence, employment status, religion, and ethnicity. The age range was 20–46 years; mean and S.D. were 29.65 and ± 4.87, respectively. Most of the respondents were graduates of tertiary educational institutions (74.1%), and all the respondents went beyond the basic primary education with 1.6% of them dropping out of secondary school. About 97% of the mothers were married while 2.3% of them were single parents. The majority of the mothers (55.7%) were employed, while about 26% of them were unemployed.

**TABLE 1:** Distribution of mothers according to age range, marital status, educational status, area of residence, employment status, religion and ethnicity.

Variables	*N*	%	Mean	s.d.
**Age (years)**				
20–25	58	18.77	29.65	± 4.87
26–35	221	71.52	-	-
36–43	25	8.09	-	-
44–46	5	1.62	-	-
**Marital status**				
Married	300	97.10	-	-
Divorced or separated	2	0.60	-	-
Single	7	2.30	-	-
**Educational status**				
Secondary school completed	30	9.70	-	-
Secondary school not completed	5	1.60	-	-
Tertiary education completed	229	74.10	-	-
Tertiary education not completed	45	14.60	-	-
**Area of residence**				
Urban area	285	92.20	-	-
Rural area	24	7.80	-	-
**Employment status**				
Employed	172	55.70	-	-
Unemployed or housewife	81	26.20	-	-
Student	56	18.10	-	-
**Religion**				
Christianity	308	99.70	-	-
Muslim	1	0.30	-	-
**Ethnicity**				
Igbo	306	99.00	-	-
Others	3	1.00	-	-

**Total**	**309**	**100**	-	-

The prevalence of post-partum anxiety was 31.1%, while that of PPD was 33.3%, and the prevalence of post-partum co-morbid anxiety and depression was 22% (See Table 2).

**TABLE 2:** Distribution of respondents according to the severity of depression and anxiety, and comorbidity between post-partum depression and anxiety.

Clinical status	Frequency	Percent	Mean	s.d.	Median	Mode
**Distribution of the mothers’ anxiety status according to HADS**						
Normal (0–7)	148	47.9	8.39	3.914	8.00	7
Mild (8–10)	68	22.0	-	-	-	-
Moderate (11–14)	67	21.7	-	-	-	-
Severe (15–21)	26	8.4	-	-	-	-
**Distribution of the mothers’ depression status according to HADS**						
Normal (0–7)	142	46.0	8.44	3.869	8.00	7
mild (8–10)	64	20.7	-	-	-	-
Moderate (11–14)	85	27.5	-	-	-	-
severe (15–21)	18	5.8	-	-	-	-
**Distribution of the mothers’ comorbidity for anxiety and depression according to HADS**						
Normal (0–7)	76	24.6	-	-	-	-
Mild –Severe (8–21)	131	42.4	-	-	-	
Moderate –Severe (11–21)	68	22.0	-	-	-	-
Severe (15–21)	6	1.94	-	-	-	-

s.d., standard deviation; HADS, Hospital Anxiety and Depression Scale.

The scores on IMS scale were not normally distributed and had a median of 22.0. Note that the IMS scores above 39 indicate marital dissatisfaction, and 39.5% (122) of the mothers had marital dissatisfaction. The distribution of the scores of women on the IMS showed that 77 of 309 (24.9%) of the women scored 42 and fell within the 75th percentile. Four (1.0%) of them had anxiety, 15 (4.9%) had depression, and 33 (10.7%) had both anxiety and depression while 25 (8.1%) of them had neither anxiety nor depression. The numbers of women with both depression and anxiety had worse marital dissatisfaction, compared to those with either anxiety or depression alone.

Table 3 shows a Spearman’s rank-order correlation coefficient, which was run to assess the relationship among PPD, post-partum anxiety and marital satisfaction. Preliminary analysis showed the relationship to be monotonic, as assessed by the visual inspection of a scatter plot. There was a moderate positive correlation between total score on IMS and HADS total score on Depression Subscale, *r*^s^ = 0.419, *p* < 0.01. There was also a moderate positive correlation between total score on IMS and total score on HADS (Anxiety Subscale), *r*^s^ = 0.401, *p* < 0.01, where *r* is the degree of freedom, obtained from the ‘Sig. (two-tailed)’ row. (Note: The higher the score on IMS, the more dissatisfaction with the relationship is indicated).

**TABLE 3:** Spearman’s rank-order correlation coefficient, assessing the relationship between post-partum depression, post-partum anxiety and marital satisfaction.

Correlations	IMS total score	HADS total score (depression subscale)	HADS total score (anxiety subscale)
**IMS total score**			
Correlation coefficient	1.000	0.419**	0.401**
*N*	309	309	309
**HADS total score (depression subscale)**			
Correlation coefficient	0.419**	1.000	0.631**
*N*	309	309	309
**HADS total score (anxiety subscale)**			
Correlation coefficient	0.401**	0.631**	1.000
*N*	309	309	309

IMS, Index of Marital Satisfaction; HADS, Hospital Anxiety and Depression Scale.

* *p* < 0.05,

** *p* < 0.01;

*** *p* < 0.001

A chi-square test for an association was conducted to examine the differences in socio-demographic data between mothers with and without PPD and PPA using the dichotomised measures of both the dependent and independent variables. The chi-square analysis found that there was a statistically significant association between educational attainment and PPD (χ^2^[1] = 7.797, *p* = 0.005) and PPA (χ^2^[1] = 8.537, *p* = 0.003).

A chi-square test for association was conducted examining the differences in socio-demographic data between mothers with and without marital satisfaction using the dichotomised measures of both the dependent and independent variables. The chi-square analysis found that there was a statistically significant association between age and marital satisfaction, (χ^2^[1] = 4.890, *p* = 0.027). There was also a statistically significant association between educational attainment and marital satisfaction (χ^2^[1] = 13.978, *p* = 0.005).

In Table 4, HADS anxiety subscale was positively related to most of the IMS items, except items 4 (I feel that I would not choose the same partner if I had to do it over), 10 (I feel that our life together is dull), 15 (I feel that we do not have enough interests in common) and 22 (I feel that we should do more things together). The strongest correlation with anxiety was found in item 12 (I feel that my partner doesn’t confide in me) (*r* = 0.37, *p* < 0.001).

**TABLE 4:** Pearson’s correlation coefficient, assessing the relationship between post-partum depression, post-partum anxiety, and 25 items of IMS.

Number	IMS items	Anxiety	Depression
1	I feel that my partner is affectionate enough	0.29***	0.18**
2	I feel that my partner treats me badly	0.16**	0.27***
3	I feel that my partner really cares for me.	0.13*	0.02
4	I feel that I would not choose the same partner if I had to do it over.	0.09	0.21***
5	I feel that I can trust my partner.	0.26***	0.28***
6	I feel that our relationship is breaking up	0.12*	0.19**
7	I feel that my partner does not understand me.	0.24***	0.40***
8	I feel that our relationship is a good one.	0.25***	0.24***
9	I feel that ours is a very happy relationship	0.27***	0.24***
10	I feel that our life together is dull	0.10	0.12*
11	I feel that we have a lot of fun together.	0.33***	0.25***
12	I feel that my partner doesn’t confide in me	0.37***	0.43***
13	I feel that ours is a very close relationship	0.27***	0.20**
14	I feel that I cannot rely on my partner	0.26***	0.38***
15	I feel that we do not have enough interests in common	−0.00	0.08
16	I feel that we manage arguments and disagreements very well	0.23***	0.26***
17	I feel that we do a good job of managing our finances	0.22***	0.17**
18	I feel that I should never have married my partner	0.14*	0.14*
19	I feel that my partner and I get along very well together	0.25***	0.29***
20	I feel that our relationship is very stable	0.26***	0.25***
21	I feel that my partner is pleased with me as a sex partner	0.17**	0.12*
22	I feel that we should do more things together	−0.01	0.06
23	I feel that the future looks bright for our relationship	0.24***	0.22***
24	I feel that our relationship is empty	0.17**	0.29***
25	I feel there is no excitement in our relationship	0.18**	0.21***

IMS, Index of Marital Satisfaction.

* *p* < 0.05,

** *p* < 0.01;

*** *p* < 0.001

The HADS Depression subscale was positively related to most of the IMS items, except items 3 (I feel that my partner really cares for me), 15 (I feel that we do not have enough interests in common) and 22 (I feel that we should do more things together). Items 4 and 22 which did not correlate significantly with anxiety were found to be significant correlates of depression. It was only item 22 that did not significantly correlate with any of the HAD subscales. The strongest correlation with depression was also found in item 12 (I feel that my partner does not confide in me) (*r* = 0.43, *p* < 0.001).

## Discussion

The study focused on women, who were in their 6th–14th weeks of postpartum, and was aimed to assess PPD and anxiety and to investigate their relationship with marital satisfaction in low-risk women. Over 74.0% of the respondents were graduates with a university degree, and all the respondents went beyond the basic primary education with only 1.6% of them dropping out of secondary school. This was a reflection of the realities of the Igbo society where high premium is, at present, placed on education, including, among the female gender, which is in line with the aphorism that said that when you educate a man, you educate a person, but when you educate a woman, you educate a complete nation; as the education of her children would be highly guaranteed; moreover, the education of every child starts from the family, and the mother is the first teacher.^49^

The majority of the mothers (55.7%) were employed, while the unemployed or housewives and students constituted 26.0% and 17.2%, respectively. As such, beyond their indispensable roles as mothers and wives, women have remarkably contributed to the building of their nation’s economy as skilled and unskilled labourers, artisans, traders, farmers, civil servants, and professionals in different areas of human endeavour.^50^ With these responsibilities, the nursing mother tries to meet the needs of her new born to adapt to the changes in her sleep pattern (most often the lack of sleep) and to recover physically from the delivery.^4^ All these factors could put the new mother at risk for psychological distress during the post-partum period.^5^

Less than 3% of the mothers were single parents; this could be related to the cultural factors primarily, as Igbo culture believes so much in strong familial ties and does not encourage single parenthood in a mother’s parental home. This aspect of Igbo culture is taken seriously, given the shame it brings to a single mother and her family, fears of parental rejection or violent reaction.^51^ In addition, there is fear of stigmatisation and discrimination from her peers and family members and friends, partner’s negative reaction or refusal to admit being responsible for the pregnancy, poor financial support, religious ostracisation, social isolation, abuse, and expulsion from school or work.^52,53^ Some factors could combine to make it very unlikely for many of such single teenage mothers to visit postnatal clinics in a tertiary hospital and could also force many of them into sheltered homes where they are taken care of, right from pregnancy to puerperium. However, Ossai and Uzochukwu,^54^ in their study, reported a higher rate (urban area = 7.8% and rural area = 10.7%) in a survey of 540 clients of maternal health service in 18 of 440 primary health care centres in urban and rural primary health care centres in Enugu state, Southeast Nigeria, who attended antenatal and postnatal clinics.

Results showed that a considerable subgroup of nursing mothers (33.3%) reported moderate-to-severe symptoms of depression; while the mothers with some possibility of having PPD (mild depression) constituted 20.7% of the mothers. Those with severe depression were 5.8%. The mean score of women’s PPD was 8.44 ± 5. 3.87. Previous research in PPD has recorded a prevalence rate of between 5.0% and 38.0%.^8,9,10,11^

Also, 35.9% of the mothers reported moderate-to-severe symptoms of anxiety; 22.0% of the mothers had mild PPA. Those with severe anxiety were 8.4%. The mean score of women’s post-partum anxiety was 8.39 ± 3.91. Previous research in PPA has recorded a prevalence rate of between 10.0% and 20.0%.^8,11^ The differences in the prevalence might be because of the effect of social, cultural, lifestyle and racial factors on depression and anxiety.^12^

The prevalence of co-morbid post-partum anxiety and depression was found to be 22.0%; previous studies had reported prevalence ranging from 2.0% to 13.0% of co-morbidity for PPD and anxiety in the first year.^55,56^

The study observed that the numbers of women with both depression and anxiety had worse marital dissatisfaction, compared to those with either anxiety or depression alone, and this is in line with report of previous studies suggesting that the mental health benefits of marriage are greater for those in ‘good-quality’ relationships in comparison to those in ‘poor-quality’ relationships.^57^

The study found an association between educational attainment and PPD and PPA. Previous studies have pointed out the role of educational attainment in the aetiology of psychological distress, and low education has been pointed out as a risk factor in mental health disorders.^58^

The study also found an association between marital satisfaction and age, and this was supported by previous studies.^59^ Also, there was an association between marital satisfaction and education attainment, and this is supported by the previous study.^60^

More importantly, this study showed that there is a significant and inverse relationship between marital satisfaction and post-partum anxiety, and this is supported by previous research involving community samples, which has shown that poor marital adjustment is associated with anxiety disorders.^34,35^ Also, in this the study, a moderate and inverse relationship between marital satisfaction and PPD was observed, and this has been reported in related previous studies elsewhere.^32,61,62^ However, O’Hara and Swain^14^ reported a small inverse relationship between poor marital relationship and PPD. Most women with PPD have lower levels of marital satisfaction and higher stress, and this may indicate the protective role of marital satisfaction in PPD^32^ and vice versa. The causal pathways between psychosocial stressors (as in degree of marital satisfaction) and depression may go in both directions.^17^Invariably, the study showed that there was a significant inverse correlation between depression, and anxiety with marital satisfaction in the post-partum period, and this has been reported in previous studies.^2,26,27,63^ Atkinson and Rickel^64^ found that, consistent with the behavioural perspective of PPD, perceived positive reinforcement was inversely related to PPD. The lack of intimacy and marital strife is linked to depression in women,^65^ and anxiety symptoms predict a decline in marital adjustment over time.^36^

The study found out in the analysis that item 12, which said: ‘I feel that my partner doesn’t confide in me’, had the strongest correlation with anxiety and depression. In a systematic review of 20 studies carried out in both rural and urban parts of Pakistan,^66^ reported the absence of a confiding relationship, as among the other four factors perceived by women to affect their mental health.^66^ This may not be out of place in Igbo culture, where the women have placed high premium on the trust of their husbands,^67^ and the husbands not confiding in their wife would most likely be perceived by a woman as because of the lack of trust and confidence in her. The perceived lack of trust and confidence could be a source of significant psychosocial distress for the women, and this could serve as a guide to areas that can be targeted in preventative programs, that will also involve couple therapy. The quality of marital relationship significantly affects the quality of mental health,^33^ and psychologically distressed individuals inadvertently contribute to the occurrence of stress in their lives, including stress in their intimate relationships, which increases the probability of the onset and maintenance of psychological distress.^68^

### Limitations of the study

The limitations of this study include the cross-sectional design (the study design should stand out clearly in the methodology), which limited the ability to draw causal conclusions. Also, this study relied exclusively on self-reported on marital satisfaction, symptoms of depression and anxiety; as such risk of inaccurate reporting may exist especially as no formal diagnostic interview was carried out. The problems that could have been associated with the pregnancy, which might have increased the psychosocial stressors, and contributions to the amount of marital disharmony experienced, were not recorded.

The researcher did not investigate the role of biological factors such as hormonal changes, psychological factors such as personality and indeed other possible factors such as substance use in determining depression and anxiety. Also, there was no adequate investigation on whether marital satisfaction caused depression and anxiety or whether depression and anxiety caused marital satisfaction, as the causal pathways may go in both directions. The inadvertent inclusion of the single mothers in the analysis might have to a slight extent, influenced the outcome of the study. As the study was conducted in two tertiary hospital populations of PPD and anxiety, it is possible that mothers with more problematic or complicated pregnancies were more than represented in the studied population. All these should be identified as significant limitations in generalising the findings, and these limitations need to be considered in future.

## Conclusion

There was a high prevalence of marital dissatisfaction, PPD and anxiety among nursing mothers in Enugu but with low detection rate. The effects of PPD and anxiety on the mother, her marital relationship and her infant make them essential conditions for early diagnosis, prevention and treatments.
